# Eligibility‐Identification Difficulty and Self‐Reported Preventive Care Implementation Among Japanese Primary Care Physicians

**DOI:** 10.1002/jgf2.70166

**Published:** 2026-08-27

**Authors:** Hironori Yamada, Kei Miyazaki, Makito Yaegashi, Kuniko Nakayama, Rei Suganaga, Nobuaki Kusaka, Wataru Hirohashi, Tomio Suzuki

**Affiliations:** ^1^ Shinjuku Tsurukame Clinic Tokyo Japan; ^2^ Department of General Medicine and General Internal Medicine Nagoya City University Graduate School of Medical Sciences Nagoya Japan; ^3^ Tokushukai Medical Corporation Tokyo Japan; ^4^ My Family Clinic Gamagori Medical Corporation MEFA Jin‐Aikai Gamagori Aichi Japan; ^5^ Kameda Family Clinic Tateyama Tateyama Chiba Japan; ^6^ Department of Postgraduate Education Center Kameda Medical Center Tateyama Chiba Japan; ^7^ Ouchi Clinic Nishiarai, Medical Corporation Daiwakai Heisei Medical Welfare Group Tokyo Japan; ^8^ Department of General Medicine, Faculty of Medicine Osaka Medical and Pharmaceutical University Takatsuki Osaka Japan

**Keywords:** health promotion, immunization, prevention, primary care, screening

## Abstract

**Background:**

Primary care is an important setting for prevention, but implementation gaps may arise before clinicians can identify patients eligible for preventive services. We described self‐reported preventive care implementation among Japanese primary care physicians and examined whether lower implementation co‐occurred with eligibility‐identification difficulty.

**Methods:**

We conducted a nationwide web‐based cross‐sectional survey of physician members of the Japan Primary Care Association from May to June 2025. Respondents reported recommendation consistency for 30 preventive services, difficulty identifying eligible patients, and perceived barriers. Service‐level implementation and eligibility‐identification difficulty were summarized in a two‐axis map.

**Results:**

Among 258 respondents, 56.2% reported structured preventive care training and 53.9% used outpatient reminders. “Mostly achieved” recommendation was relatively high for hypertension, diabetes, and dyslipidaemia screening and influenza vaccination, but low for screening for domestic violence, chlamydia/gonorrhea, abdominal aortic aneurysm, anxiety, and depression, respiratory syncytial virus vaccination, and folic acid counseling. Difficulty identifying eligible patients was highest for domestic violence screening (14.0%), chlamydia/gonorrhea screening (13.6%), hepatitis C screening (10.5%), and respiratory syncytial virus vaccination (7.8%). Common barriers were time constraints, insufficient knowledge or skills, low patient interest, and financial or reimbursement issues.

**Conclusions:**

Self‐reported implementation varied substantially across services. Some low‐implementation services also involved eligibility‐identification difficulty, suggesting an upstream implementation problem. Japanese primary care may need systems that make eligibility visible through training, reminders, prompts, registries, and team‐based workflows.

## 1 Introduction

Primary care is a key setting for prevention because it provides continuing, comprehensive, and accessible care across the life course [1]. Preventive care in this setting includes vaccination, cancer screening, cardiometabolic screening, behavioral counseling, and other activities that require both evidence‐informed recommendations and workable clinical systems [2]. However, integrating prevention into routine practice remains difficult. Preventive services compete with acute care, chronic disease management, administrative work, and patient priorities during time‐limited consultations. Previous estimates suggest that delivering recommended adult preventive services alone would require substantial physician time and that providing preventive, chronic, acute, and administrative care for a representative adult panel would exceed the time available to an individual primary care physician without team‐based support [3, 4].

Evidence from Japan suggests that primary care provides opportunities for prevention, but important gaps remain. A nationwide Japanese study reported that having a usual source of primary care was associated with patient‐reported receipt of several preventive care measures, while receipt remained low for some services such as depression screening, zoster vaccination, and tetanus vaccination [5]. These findings suggest that access to primary care alone may not ensure consistent preventive care implementation.

Most studies describe whether preventive services are delivered or received. Less attention has been paid to an earlier implementation step: whether clinicians can identify which patients are eligible for recommendation. Eligibility may depend on age, sex, pregnancy potential, sexual exposure, vaccination history, medical risk, family history, previous screening results, municipal subsidy systems, or occupational health check pathways. When eligibility is not recognized during routine care, gaps may appear as low implementation even before a recommendation decision is made. Reminder systems, clinical decision support, registries, checklists, and team‐based workflows may therefore be important not only as prompts to recommend care but also as infrastructure for identifying eligible patients [6, 7, 8].

We conducted a nationwide web‐based survey of physician members of the Japan Primary Care Association. We assessed self‐reported recommendation of 30 preventive services and mapped each service according to two dimensions: the percentage of respondents reporting “mostly achieved” recommendation and the percentage reporting inability to identify eligible patients. The aim was to examine whether some preventive care gaps in Japanese primary care involve an earlier step: recognizing who is eligible for recommendation.

## 2 Methods

### 2.1 Study Design and Setting

This was a nationwide web‐based cross‐sectional survey of physician members of the Japan Primary Care Association. The survey was conducted using Google Forms from May to June 2025. The study is reported in accordance with the Strengthening the Reporting of Observational Studies in Epidemiology guidance for cross‐sectional studies [9].

### 2.2 Participants and Recruitment

Eligible participants were physician members of the Japan Primary Care Association. Survey invitations were distributed through the association mailing list to physician members registered on the list. According to administrative data provided by the association, 6369 physician members were registered on the mailing list as of June 2, 2025, the closest available registration count to survey commencement. Participation was voluntary. Respondents provided electronic informed consent before answering the questionnaire. Responses were anonymous; names, contact details, email addresses, Google account information, and internet protocol addresses were not collected. Respondents were instructed to answer only once, although duplicate responses could not be technically prevented.

### 2.3 Survey Instrument and Variables

The questionnaire was developed for this study with reference to the scope of clinical preventive services in primary care. It included respondent characteristics, practice environment, structured preventive care training, teaching or lecturing experience, reminder use, preventive care resources, self‐reported recommendation of preventive services during the previous year, perceived barriers, patient‐level reasons for non‐receipt as perceived by respondents, support needs, future priority domains, perceived need for team‐based care, and free‐text comments.

Self‐reported implementation was assessed for 30 preventive services: five cancer screening services, nine vaccination services, 11 screening services, and five counseling services. For each service, respondents were asked how well they had appropriately recommended the service to patients whom they considered to need it as preventive care during the previous year. “Appropriately recommended” was based on respondents' own clinical judgment informed by prior learning and experience, rather than strict adherence to any single guideline. Response options were “unable to identify eligible patients,” “no eligible patients,” “not at all achieved,” “rarely achieved (0%–30%),” “moderately achieved (40%–60%),” and “mostly achieved (70%–100%).”

### 2.4 Outcome and Definitions

The primary item‐level outcome was the percentage reporting “mostly achieved.” Respondents selecting “no eligible patients” for that service were excluded from the denominator. A secondary item‐level outcome was “good/moderate achievement,” defined as “moderately achieved” or “mostly achieved,” again excluding respondents selecting “no eligible patients” from the denominator.

Difficulty identifying eligible patients was defined as the percentage selecting “unable to identify eligible patients,” using all valid responses for that service as the denominator. The percentage selecting “no eligible patients” also used all valid responses for that service as the denominator.

A two‐axis eligibility‐identification map was created to examine whether lower self‐reported recommendation achievement co‐occurred with difficulty identifying eligible patients. The x‐axis represented the percentage of respondents selecting “unable to identify eligible patients” for each preventive service, and the y‐axis represented the percentage selecting “mostly achieved” regarding appropriate recommendation of that service. The median value of each axis across the 30 services was used as a descriptive reference line. We defined the lower‐right quadrant, where difficulty identifying eligible patients was at or above the median and self‐reported recommendation achievement was below the median, as the eligibility‐identification priority zone. This classification was exploratory and intended to describe implementation patterns rather than rank clinical importance.

Separately, a respondent‐level composite implementation score was calculated as the percentage of services rated “moderately achieved” or “mostly achieved,” excluding services for which the respondent reported “no eligible patients” and retaining “unable to identify eligible patients” in the denominator as not achieved.

### 2.5 Statistical Analysis

Descriptive statistics were used throughout. Categorical variables are reported as numbers and percentages. Continuous variables are reported as means with standard deviations and medians with interquartile ranges. Item‐level preventive care measures were summarized using the denominator definitions described above. Ninety‐five per cent confidence intervals for item‐level percentages were calculated using the Wilson score method.

Sensitivity analyses using alternative priority‐zone definitions were conducted. Exploratory descriptive comparisons of the respondent‐level composite implementation score and overall self‐rated implementation score were conducted by training, teaching experience, reminder use, resource use, years in practice, workplace, and age group. An exploratory multivariable linear regression model examined factors associated with the composite implementation score using HC3 heteroscedasticity‐robust standard errors. These exploratory analyses were not interpreted causally, and no adjustment for multiple comparisons was applied.

Free‐text comments were coded thematically by multiple authors, with themes discussed and refined by the author group. Comments could be assigned to more than one theme.

## 3 Results

### 3.1 Respondent Characteristics and Practice Environment

A total of 258 valid responses were included. According to the association's administrative records, 6369 physician members were registered on the mailing list on June 2, 2025, the closest available count to survey commencement; the 258 valid responses therefore corresponded to 4.1% of this mailing‐list registration count. Because the registration count did not verify the number of unique members to whom the invitation was successfully delivered, this crude proportion was not treated as a conventional response rate. The mean age was 48.6 years, 74.0% were male, and 41.9% worked primarily in clinics. General medicine was the most frequently reported specialty. Structured preventive care training was reported by 56.2%, teaching or lecturing experience by 38.0%, and reminder use in regular outpatient care by 53.9%. Most respondents used at least one preventive care resource, while 30 respondents selected only “few or no resources.” The mean overall self‐rated implementation score was 48.6 out of 100 (Table 1). Detailed respondent characteristics and practice environment are provided in Table S1.

**TABLE 1:** Characteristics and practice environment of 258 Japan Primary Care Association physician respondents, Japan, 2025.

Characteristic	Category or statistic	*n* (%) or value
Age, years	Mean (standard deviation)	48.6 (11.8)
	Median (interquartile range)	48.0 (39.0–57.0)
Age group	≤ 44 years	93 (36.0)
	45–54 years	82 (31.8)
	≥ 55 years	83 (32.2)
Sex	Male	191 (74.0)
	Female	62 (24.0)
	Other, selected both, or prefer not to answer	5 (1.9)
Years in practice	< 10 years	40 (15.5)
	10 to < 30 years	157 (60.9)
	≥ 30 years	61 (23.6)
Primary workplace	Clinic	108 (41.9)
	Small‐ to medium‐sized hospital	69 (26.7)
	University hospital	42 (16.3)
	Regional core hospital	34 (13.2)
	Other	5 (1.9)
Main clinical setting	Outpatient care	155 (60.1)
	Inpatient care	74 (28.7)
	Home or facility visits	20 (7.8)
	Other	9 (3.5)
Employment status	Full‐time physician	218 (84.5)
	Part‐time physician	21 (8.1)
	Resident	16 (6.2)
	Other	3 (1.2)
Specialty	General medicine	189 (73.3)
	Internal medicine	137 (53.1)
	Pediatrics	31 (12.0)
	Surgery	9 (3.5)
	Rehabilitation medicine	9 (3.5)
	Emergency medicine	9 (3.5)
	Orthopedic surgery	8 (3.1)
	Psychiatry	5 (1.9)
	Other specialties (≥ 1 specialty selected by < 5 respondents)	28 (10.9)
Structured preventive care training	Yes	145 (56.2)
Preventive care teaching or lecturing experience	Yes	98 (38.0)
Reminder use in regular outpatient care	Yes	139 (53.9)
Preventive care resources	Any resource used	228 (88.4)
	Few or no resources only	30 (11.6)
Outpatient preventive care activity	≥ 31% of patients	119 (46.1)
Inpatient preventive care activity	Not engaged in inpatient care	121 (46.9)
	≥ 31% of inpatients	30 (11.6)
Overall self‐rated implementation score	Mean (standard deviation); *n* = 256	48.6 (24.0)
	Median (interquartile range); *n* = 256	50.0 (30.0–70.0)

*Note:* Percentages use *N* = 258 unless otherwise indicated. Specialty was a multiple‐response item. “Any resource used” includes respondents who selected at least one preventive care resource; “few or no resources only” includes respondents selecting only the “few or no resources” option. Overall self‐rated implementation was summarized among respondents with numeric responses. The terms “preventive care‐related activity” and “self‐rated implementation” refer to respondent self‐report and do not indicate documented clinical delivery or patient receipt.

### 3.2 Variation in Self‐Reported Preventive Care Implementation

Self‐reported implementation varied substantially across services. Cardiometabolic screening had relatively high “mostly achieved” percentages: hypertension screening 49.2%, diabetes screening 49.0%, and dyslipidaemia screening 47.5%. Influenza vaccination was also relatively high at 49.2%. Good/moderate achievement exceeded 85% for hypertension, diabetes, and dyslipidaemia screening and influenza vaccination.

Lower “mostly achieved” percentages were observed for domestic violence screening (4.4%), chlamydia/gonorrhea screening (6.0%), respiratory syncytial virus vaccination (6.2%), folic acid supplementation counseling (6.4%), abdominal aortic aneurysm screening (7.6%), anxiety screening (11.2%), depression screening (13.2%), breast cancer screening (14.6%), cervical cancer screening (17.0%), and selected adult vaccinations. Detailed item‐level results for all 30 services are provided in Table S2, with 95% confidence intervals in Table S3, and displayed in Figure S1.

### 3.3 Difficulty Identifying Eligible Patients and the Priority Zone

The median unable‐to‐identify percentage across services was 3.10%, and the median mostly‐achieved percentage was 21.08%. Difficulty identifying eligible patients was highest for domestic violence screening (14.0%), chlamydia/gonorrhea screening (13.6%), hepatitis C screening (10.5%), respiratory syncytial virus vaccination (7.8%), hepatitis B vaccination (6.6%), folic acid supplementation counseling (5.4%), anxiety screening (5.4%), and abdominal aortic aneurysm screening (5.0%).

**FIGURE 1:**
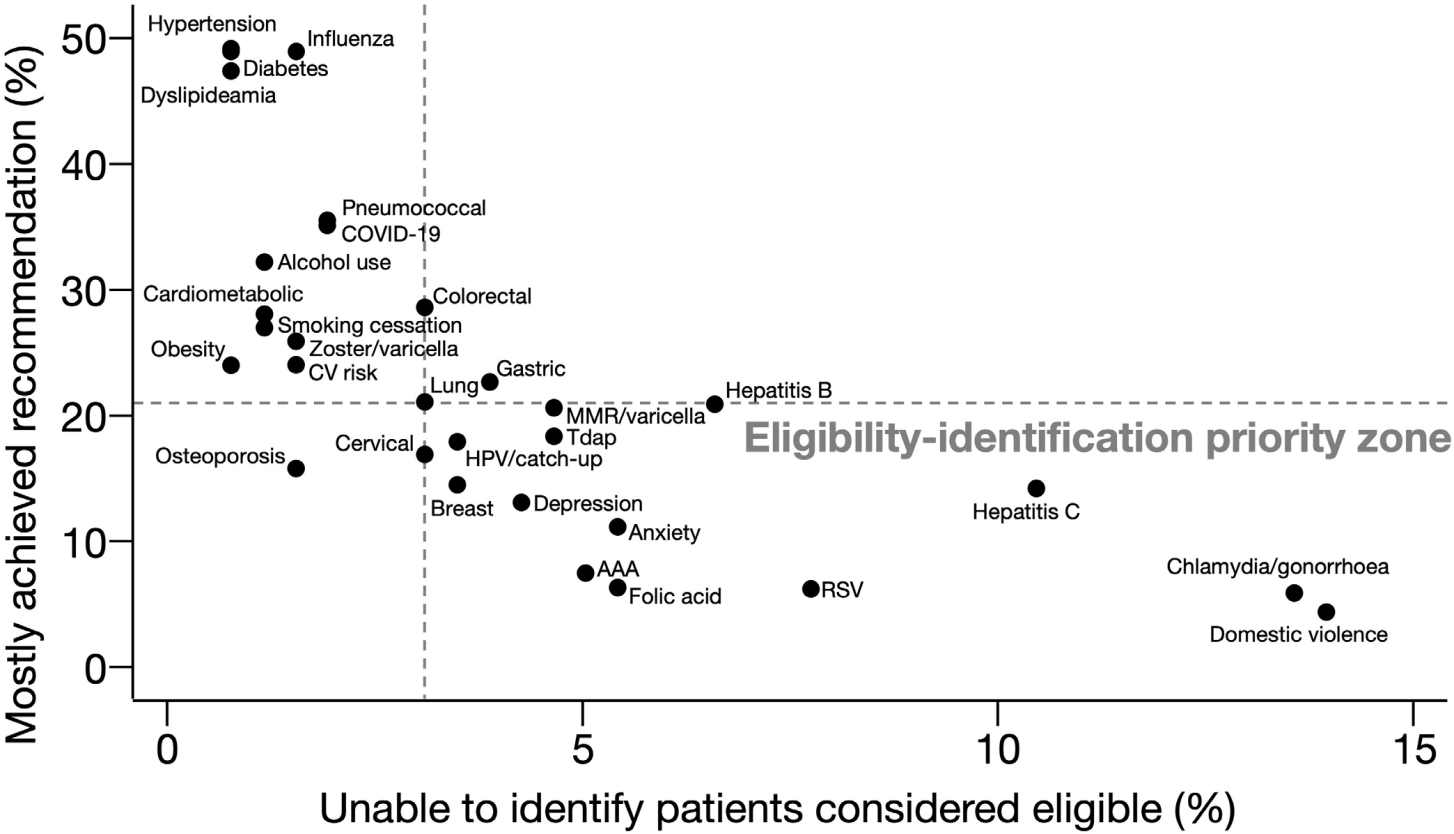
Two‐axis map of preventive services reported by 258 Japan Primary Care Association physician respondents, Japan, 2025. Legend: Each point represents one preventive service. The x‐axis shows the percentage of valid responses selecting “unable to identify eligible patients.” The *y*‐axis shows the percentage selecting “mostly achieved (70%–100%),” excluding respondents selecting “no eligible patients” from the denominator. Dashed lines show median values across the 30 services: 3.10% for difficulty identifying eligible patients and 21.08% for mostly achieved recommendation. The priority zone was defined descriptively and exploratorily as services at or above the median for difficulty identifying eligible patients and below the median for mostly achieved recommendation. AAA, abdominal aortic aneurysm; COVID‐19, coronavirus disease 2019; CV risk, cardiovascular risk; HPV, human papillomavirus; MMR, measles, mumps and rubella; RSV, respiratory syncytial virus; Tdap, tetanus, diphtheria and pertussis.

The two‐axis map identified 14 services in the eligibility‐identification priority zone: breast cancer screening, cervical cancer screening, tetanus/diphtheria/pertussis vaccination, measles/mumps/rubella/varicella vaccination, human papillomavirus and catch‐up human papillomavirus vaccination, hepatitis B vaccination, respiratory syncytial virus vaccination, chlamydia/gonorrhea screening, hepatitis C screening, abdominal aortic aneurysm screening, anxiety screening, depression screening, domestic violence screening, and folic acid supplementation counseling (Figure 1 and Table 2). According to the predefined descriptive classification, these services had below‐median self‐reported recommendation and at‐or‐above‐median difficulty identifying eligible patients. Osteoporosis screening was the only service classified in the lower‐difficulty/lower‐achievement quadrant, with 15.9% reporting mostly achieved recommendation and 1.6% reporting inability to identify eligible patients. The full quadrant classification is shown in Table S4, and sensitivity analyses using alternative priority‐zone definitions are shown in Table S5.

**TABLE 2:** Preventive services in the eligibility‐identification priority zone among 258 Japan Primary Care Association physician respondents, Japan, 2025.

Domain	Preventive service	Mostly achieved, *n*/*N* (%)	Good/moderate achievement, *n*/*N* (%)	Unable to identify eligible patients, *n*/*N* (%)	No eligible patients, *n*/*N* (%)
Cancer screening	Breast cancer screening	33/226 (14.6)	96/226 (42.5)	9/258 (3.5)	32/258 (12.4)
Cancer screening	Cervical cancer screening	38/224 (17.0)	99/224 (44.2)	8/258 (3.1)	34/258 (13.2)
Vaccination	Tetanus, diphtheria and pertussis vaccination	40/217 (18.4)	80/217 (36.9)	12/258 (4.7)	41/258 (15.9)
Vaccination	Measles, mumps, rubella and varicella vaccination	45/218 (20.6)	94/218 (43.1)	12/258 (4.7)	40/258 (15.5)
Vaccination	Human papillomavirus and catch‐up human papillomavirus vaccination	38/211 (18.0)	92/211 (43.6)	9/258 (3.5)	47/258 (18.2)
Vaccination	Hepatitis B vaccination	46/219 (21.0)	91/219 (41.6)	17/258 (6.6)	39/258 (15.1)
Vaccination	Respiratory syncytial virus vaccination	13/209 (6.2)	25/209 (12.0)	20/258 (7.8)	49/258 (19.0)
Screening	Chlamydia/gonorrhea screening	12/201 (6.0)	29/201 (14.4)	35/258 (13.6)	57/258 (22.1)
Screening	Hepatitis C screening	32/225 (14.2)	94/225 (41.8)	27/258 (10.5)	33/258 (12.8)
Screening	Abdominal aortic aneurysm screening	18/238 (7.6)	68/238 (28.6)	13/258 (5.0)	20/258 (7.8)
Screening	Anxiety screening	28/250 (11.2)	76/250 (30.4)	14/258 (5.4)	8/258 (3.1)
Screening	Depression screening	33/250 (13.2)	80/250 (32.0)	11/258 (4.3)	8/258 (3.1)
Screening	Domestic violence screening	10/226 (4.4)	25/226 (11.1)	36/258 (14.0)	32/258 (12.4)
Counseling	Folic acid supplementation counseling	14/218 (6.4)	47/218 (21.6)	14/258 (5.4)	40/258 (15.5)

*Note:* “Mostly achieved” corresponds to 70%–100% achieved. “Good/moderate achievement” combines 40%–60% and 70%–100% achieved. For both achievement measures, respondents selecting “no eligible patients” were excluded from the denominator. “Unable to identify eligible patients” and “no eligible patients” use all valid responses for that preventive service as denominators. The priority zone was defined descriptively and exploratorily as services with unable‐to‐identify percentage at or above the median across services and mostly‐achieved percentage below the median.

### 3.4 Perceived Barriers, Patient‐Level Reasons, and Support Needs

The most frequently reported barriers were time constraints (72.5%), lack of knowledge or skills among healthcare professionals (54.7%), low patient interest (50.4%), and financial or reimbursement‐related issues (45.3%). Respondents perceived insufficient patient awareness of prevention (82.6%), financial burden (62.0%), and time constraints (57.4%) as common patient‐level reasons for not receiving preventive care.

The most frequently selected support needs were training and education opportunities (66.3%), financial support for preventive care activities (58.5%), team‐based care with other professionals (53.9%), and development or revision of practical guidelines (51.2%). Vaccination was the most frequently selected future priority domain (66.7%), followed by cancer screening (49.6%) and screening other than cancer screening (47.7%) (Table 3). In a separate item, 98.1% of respondents regarded team‐based preventive care as at least somewhat necessary.

**TABLE 3:** Reported barriers, perceived patient‐level reasons, support needs and priority domains among 258 Japan Primary Care Association physician respondents, Japan, 2025.

Category	Item	*n* (%)
Barriers to preventive care	Time constraints	187 (72.5)
	Lack of knowledge or skills among healthcare professionals	141 (54.7)
	Low patient interest	130 (50.4)
	Financial or reimbursement‐related issues	117 (45.3)
	Insufficient organizational support	61 (23.6)
	Lack of collaboration with administrative sectors	54 (20.9)
	Insufficient equipment or infrastructure	46 (17.8)
Patient‐level reasons perceived by respondents	Insufficient awareness of the need for prevention	213 (82.6)
	Financial burden	160 (62.0)
	Time constraints	148 (57.4)
	Difficulty understanding insurance or public subsidy systems	107 (41.5)
	Anxiety or fear, including fear of test results	81 (31.4)
Support needed for implementation	Training and education opportunities	171 (66.3)
	Financial support for preventive care activities	151 (58.5)
	Team‐based care with other professionals	139 (53.9)
	Development or revision of practical guidelines	132 (51.2)
Future priority domains	Vaccination	172 (66.7)
	Cancer screening	128 (49.6)
	Screening other than cancer screening	123 (47.7)
	Counseling	69 (26.7)
	No particular priority domain	24 (9.3)

*Note:* Percentages use *N* = 258. Items shown in this table were multiple‐response items. Patient‐level reasons are respondents' perceptions and were not directly reported by patients. The full response distribution for the separate item on perceived need for team‐based preventive care is reported in Table S1.

### 3.5 Exploratory Comparisons

The mean respondent‐level composite implementation score was 55.1% (standard deviation 25.4). In exploratory comparisons, respondents with structured preventive care training, reminder use, and use of preventive care resources reported higher composite implementation scores and higher overall self‐rated implementation scores than their counterparts without these supports. In exploratory multivariable analysis, structured training and reminder use were associated with higher composite implementation scores. These analyses are reported in Tables S6 and S7 and should not be interpreted causally. Themes from free‐text comments are presented in Table S8.

## 4 Discussion

### 4.1 Principal Findings

In this nationwide web‐based survey of physician members of the Japan Primary Care Association, self‐reported preventive care implementation varied widely across 30 preventive services. Cardiometabolic screening and influenza vaccination appeared relatively embedded in routine practice, whereas services related to sexual health, mental health, domestic violence, reproductive health, and selected adult vaccinations were less consistently reported. Several lower‐achievement services were also services for which respondents reported difficulty identifying eligible patients. This points to an implementation problem that can occur before a recommendation is made: recognizing who should be considered eligible.

### 4.2 Interpretation

Services tied to routine adult primary care tended to have higher reported achievement. Hypertension, diabetes, and dyslipidaemia screening are closely linked to chronic disease management and to legally mandated or publicly endorsed health check systems in Japan. Japanese primary care physicians have also been reported to perceive publicly endorsed periodic health examinations as effective [10]. This institutionalization may make both eligibility and action more visible in routine practice. Influenza vaccination is familiar, seasonal, and supported by public awareness, and recent Japanese evidence links better patient experience in primary care with higher influenza vaccine uptake [11]. These services were outside the eligibility‐identification priority zone because respondents reported relatively high achievement and relatively little difficulty identifying eligible patients.

The eligibility‐identification priority zone was different. It contained services for which eligibility may be less visible during ordinary consultations. Selected adult vaccinations, chlamydia/gonorrhea screening, hepatitis C screening, abdominal aortic aneurysm screening, anxiety and depression screening, domestic violence screening, and folic acid supplementation counseling require information that is not always routinely captured. Eligibility may depend on past vaccination records, sexual history, pregnancy potential, age‐sex‐risk combinations, previous screening results, family or social circumstances, or sensitive information that patients may not volunteer without a suitable clinical process. For these services, increasing recommendations may be difficult unless practices can first make eligibility visible.

The highest unable‐to‐identify percentage was 14.0%, so the absolute level of reported difficulty should not be overstated. Still, this response option captures a specific problem: the respondent could not readily determine who should be considered eligible for that service. When this difficulty is combined with low self‐reported achievement, the service may not yet be operationalized as a routine, recognizable task within the practice. The priority map should therefore be interpreted not as a ranking of clinical importance, but as a descriptive tool for distinguishing services that may require eligibility‐identification support from those requiring other forms of implementation support.

Preventive care is rarely completed in a single consultation. It requires prioritization, sequencing, explanation, follow‐up, and repeated opportunities for recommendation [3, 4]. Screening is also a process rather than a test alone: positive results require explanation, further assessment, and decisions that balance benefits and harms [12]. General health checks and optional screening may create additional interpretation and follow‐up needs, and their benefits and harms should not be assumed to be uniformly favorable [13, 14]. In Japan, cancer screening, occupational health checks, specific health checkups, optional comprehensive health checks, and routine clinical care are organized through multiple pathways. This fragmented structure may make it difficult for primary care practices to know what has already been done, what remains indicated, and who is responsible for follow‐up [15, 16].

This interpretation aligns with previous literature suggesting that preventive care implementation depends on organizational context, reminders, education, and multicomponent strategies rather than clinician intention alone [6, 7, 8]. In our exploratory analyses, respondents with structured preventive care training or reminder use reported higher composite implementation scores. These associations should not be interpreted causally. They nevertheless support treating training, reminders, practical resources, electronic prompts, checklists, registries, and team‐based workflows as practice supports that can help identify eligible patients, distribute preventive care tasks, provide consistent advice, and reduce reliance on individual physician memory during time‐limited encounters.

### 4.3 Implications for Practice and Research

The map may help practices separate two problems. Services with low self‐reported achievement but low reported difficulty identifying eligible patients may require knowledge translation, patient engagement, reimbursement support, or workflow redesign after eligibility is already recognized. Services with both low self‐reported achievement and higher reported difficulty identifying eligible patients may need tools that bring eligibility into view, such as structured intake forms, vaccination history tools, electronic health record prompts, registries, recall systems, preventive care checklists, and team‐based workflows.

Osteoporosis screening illustrated the former pattern. In our study, osteoporosis screening was the only service classified in the lower‐difficulty/lower‐achievement quadrant: 15.9% of respondents reported mostly achieved recommendation, whereas only 1.6% reported being unable to identify eligible patients. This pattern is consistent with previous reports suggesting low uptake of organized osteoporosis screening in Japan. A report on osteoporosis screening under the Health Promotion Act noted that 287,782 women underwent screening in 2008, corresponding to 4.7% of the estimated eligible female population [17]. More recent national statistics reported 319,819 osteoporosis screening recipients in fiscal year 2023, with screening implemented by 1115 municipalities, corresponding to 64.2% of municipalities [18]. These data suggest that the implementation gap for osteoporosis screening may arise less from identifying eligible patients and more from downstream steps after eligibility recognition, including translating risk recognition into an explicit recommendation, arranging bone‐density assessment, and addressing patient preferences, cost, access, or competing priorities. Because this survey did not collect item‐specific reasons for non‐implementation, this interpretation should be regarded as hypothesis‐generating.

For domestic violence, sexual health, mental health, and reproductive health, eligibility identification also requires attention to confidentiality, stigma, communication skills, and clinic processes that allow sensitive enquiry. For adult vaccination, implementation may depend less on memorizing every vaccine recommendation and more on establishing reliable triggers: who should check vaccination history, when reminders should appear, and which team member should provide information or arrange follow‐up. These are system design questions rather than problems of individual physician effort alone.

Future research should link physician‐reported implementation with clinical record data, patient‐reported receipt, and practice‐level infrastructure. Qualitative studies could clarify how clinicians judge eligibility when national recommendations, municipal subsidy systems, insurance coverage, occupational health checks, and patient preferences are not well aligned. Intervention studies could examine whether eligibility‐identification tools embedded in electronic health records, adult preventive care checklists, nurse‐ or pharmacist‐led workflows, and recall systems are feasible and acceptable in Japanese primary care.

### 4.4 Strengths and Limitations

This study has several strengths. It assessed 30 preventive services using the same response framework and explicitly separated self‐reported recommendation from difficulty identifying eligible patients. The two‐axis map provides an implementation‐oriented view of preventive care gaps and highlights services for which eligibility identification itself may be a barrier. The survey also captured barriers, support needs, perceived patient‐level reasons, and free‐text comments, allowing quantitative findings to be interpreted in relation to practice infrastructure.

Several limitations should be noted. First, this voluntary web‐based survey may have been affected by substantial selection and nonresponse bias; physicians with greater interest in preventive care may have been more likely to respond, potentially leading to overestimation of implementation. The 258 valid responses corresponded to 4.1% of the 6369 physician members registered on the association mailing list on June 2, 2025. Because this mailing‐list registration count was not a verified count of unique members to whom the invitation was successfully delivered, this crude proportion should not be interpreted as a conventional response rate. The participation proportion among members to whom the invitation was successfully delivered may therefore have been higher than 4.1%, but could not be estimated reliably. Second, aggregate demographic and practice data for physician members registered on the mailing list were unavailable to the study team, so respondent representativeness within the mailing‐list population could not be assessed; moreover, the findings may not represent all physicians providing primary care in Japan. Third, all outcomes were self‐reported and may be affected by recall bias and social desirability bias. Fourth, the survey measured self‐reported recommendation rather than actual clinical behavior, documented recommendation, patient receipt of preventive services, or clinical outcomes. Fifth, the questionnaire was developed for this study and was not a previously validated instrument. Sixth, the study was cross‐sectional, so associations with training, reminder use, resources, and practice environment cannot be interpreted causally. Seventh, the priority zone was a descriptive and exploratory classification based on medians across services; it should be used to generate implementation hypotheses rather than to rank services definitively. Eighth, respondent judgment was used to define appropriate recommendation and eligibility, so the findings should not be interpreted as a strict assessment of clinical practice guideline‐concordant care. Ninth, respondents' perceptions of patient‐level reasons were not directly reported by patients. Finally, duplicate responses could not be technically prevented because the survey was anonymous, although respondents were instructed to answer only once.

## 5 Conclusion

Self‐reported preventive care implementation among primary care physicians in Japan varied substantially across services. Cardiometabolic screening and influenza vaccination were more consistently reported, whereas sexual health, mental health, domestic violence, reproductive health, and selected adult vaccination services showed lower self‐reported implementation. For several low‐achievement services, respondents also reported difficulty identifying eligible patients. Educational interventions alone may not be sufficient; preventive care support may also need eligibility‐identification tools, reminders, practical resources, electronic prompts, checklists, registries, and team‐based workflows.

## Author Contributions

**Kei Miyazaki:** conceptualization, methodology, project administration, writing – review and editing, supervision. **Rei Suganaga:** conceptualization, methodology, project administration, writing – review and editing. **Nobuaki Kusaka:** conceptualization, project administration, writing – original draft. **Makito Yaegashi:** conceptualization, methodology, project administration, writing – review and editing, supervision. **Wataru Hirohashi:** conceptualization, project administration, writing – review and editing. **Kuniko Nakayama:** conceptualization, methodology, supervision, project administration, writing – review and editing. **Hironori Yamada:** conceptualization, methodology, software, data curation, formal analysis, visualization, investigation, writing – original draft, writing – review and editing, resources, project administration. **Tomio Suzuki:** conceptualization, methodology, investigation, supervision, funding acquisition, project administration, writing – review and editing.

## Funding

This research received no specific grant from any funding agency in the public, commercial, or not‐for‐profit sectors.

## Ethics Statement

This study was approved by the Ethics Committee of the Japan Primary Care Association. Approval number: 2025‐054.

## Consent

Participation in the survey was voluntary. Responses were collected anonymously, and informed consent was obtained electronically before participation.

## Conflicts of Interest

The authors declare no conflicts of interest.

## Supporting information


**Figure S1:** Item‐level self‐reported recommendation and difficulty identifying eligible patients for 30 preventive services. Bars show the percentage of respondents reporting “mostly achieved (70%–100%).” Circles show the percentage reporting “unable to identify eligible patients.” “Mostly achieved” excludes respondents selecting “no eligible patients” from the denominator. “Unable to identify eligible patients” uses all valid responses for each service as the denominator. The figure summarizes physician self‐reported recommendation and does not indicate documented clinical delivery or patient receipt. AAA, abdominal aortic aneurysm; COVID‐19, coronavirus disease 2019; CV risk, cardiovascular risk; HPV, human papillomavirus; MMR, measles, mumps and rubella; RSV, respiratory syncytial virus; Tdap, tetanus, diphtheria and pertussis.
**Table S1:** Full respondent characteristics and detailed practice environment.
**Table S2:** Self‐reported recommendation and eligibility‐identification difficulty for 30 preventive services.
**Table S3:** Item‐level percentages with 95% confidence intervals for 30 preventive services.
**Table S4:** Quadrant classification for the two‐axis priority map.
**Table S5:** Sensitivity analyses for alternative priority‐zone definitions.
**Table S6:** Exploratory descriptive comparisons of respondent‐level implementation scores.
**Table S7:** Exploratory multivariable analysis of the respondent‐level composite implementation score.
**Table S8:** Themes from free‐text comments with anonymised illustrative quotations.

## Data Availability

The data that support the findings of this study are available on request from the corresponding author. The data are not publicly available due to privacy or ethical restrictions.
