# Supplementary material for: Eligibility‐Identification Difficulty and Self‐Reported Preventive Care Implementation Among Japanese Primary Care Physicians
Source: J Gen Fam Med. 2026 Aug 27;27(5):e70166. doi: 10.1002/jgf2.70166 (PMC13522630; doi:10.1002/jgf2.70166)
Supplement: Supplementary file 1 [file JGF2-27-e70166-s001.docx]

Supplementary Material

**Eligibility-identification difficulty and self-reported preventive care implementation among Japanese primary care physicians**

Definitions used throughout the supplementary material:

“Mostly achieved” corresponds to 70–100% achieved. “Good/moderate achievement” combines 40–60% and 70–100% achieved. Both achievement measures exclude respondents who selected “no eligible patients” from the denominator. “Unable to identify eligible patients” and “no eligible patients” use all valid responses for each service as denominators. The priority-zone classification is descriptive and exploratory. All preventive care measures refer to physician self-reported recommendation and should not be interpreted as documented clinical delivery, patient receipt, or guideline-concordant care.

*Figure*

Supplementary Figure S1. Item-level self-reported recommendation and difficulty identifying eligible patients for 30 preventive services.

Bars show the percentage of respondents reporting “mostly achieved (70–100%).” Circles show the percentage reporting “unable to identify eligible patients.” “Mostly achieved” excludes respondents selecting “no eligible patients” from the denominator. “Unable to identify eligible patients” uses all valid responses for each service as the denominator. The figure summarises physician self-reported recommendation and does not indicate documented clinical delivery or patient receipt.

Abbreviations: AAA, abdominal aortic aneurysm; COVID-19, coronavirus disease 2019; CV risk, cardiovascular risk; HPV, human papillomavirus; MMR, measles, mumps and rubella; RSV, respiratory syncytial virus; Tdap, tetanus, diphtheria and pertussis.

*Tables*

Supplementary Table S1. Full respondent characteristics and detailed practice environment.

| **Section** | **Characteristic** | **Category or statistic** | **n (%) or value** |
| --- | --- | --- | --- |
| Respondent characteristics | Age, years | Mean (standard deviation) | 48.6 (11.8) |
| Respondent characteristics |  | Median (interquartile range) | 48.0 (39.0–57.0) |
| Respondent characteristics | Age group | ≤34 years | 33 (12.8) |
| Respondent characteristics |  | 35–44 years | 60 (23.3) |
| Respondent characteristics |  | 45–54 years | 82 (31.8) |
| Respondent characteristics |  | 55–64 years | 61 (23.6) |
| Respondent characteristics |  | ≥65 years | 22 (8.5) |
| Respondent characteristics | Sex | Male | 191 (74.0) |
| Respondent characteristics |  | Female | 62 (24.0) |
| Respondent characteristics |  | Other / selected both / prefer not to answer | 5 (1.9) |
| Respondent characteristics | Years in practice | <5 years | 19 (7.4) |
| Respondent characteristics |  | 5 to <10 years | 21 (8.1) |
| Respondent characteristics |  | 10 to <20 years | 77 (29.8) |
| Respondent characteristics |  | 20 to <30 years | 80 (31.0) |
| Respondent characteristics |  | ≥30 years | 61 (23.6) |
| Respondent characteristics | Primary workplace | University hospital | 42 (16.3) |
| Respondent characteristics |  | Regional core hospital | 34 (13.2) |
| Respondent characteristics |  | Small- to medium-sized hospital | 69 (26.7) |
| Respondent characteristics |  | Clinic | 108 (41.9) |
| Respondent characteristics |  | Other | 5 (1.9) |
| Respondent characteristics | Main clinical setting | Outpatient care | 155 (60.1) |
| Respondent characteristics |  | Inpatient care | 74 (28.7) |
| Respondent characteristics |  | Home or facility visits | 20 (7.8) |
| Respondent characteristics |  | Other | 9 (3.5) |
| Respondent characteristics | Employment status | Full-time physician | 218 (84.5) |
| Respondent characteristics |  | Part-time physician | 21 (8.1) |
| Respondent characteristics |  | Resident | 16 (6.2) |
| Respondent characteristics |  | Other | 3 (1.2) |
| Respondent characteristics | Specialty† | General medicine | 189 (73.3) |
| Respondent characteristics |  | Internal medicine | 137 (53.1) |
| Respondent characteristics |  | Paediatrics | 31 (12.0) |
| Respondent characteristics |  | Surgery | 9 (3.5) |
| Respondent characteristics |  | Rehabilitation medicine | 9 (3.5) |
| Respondent characteristics |  | Emergency medicine | 9 (3.5) |
| Respondent characteristics |  | Orthopaedic surgery | 8 (3.1) |
| Respondent characteristics |  | Psychiatry | 5 (1.9) |
| Respondent characteristics |  | Other specialties (≥1 specialty selected by <5 respondents) | 28 (10.9) |
| Preventive care experience and practice environment | Structured training in preventive care | Yes | 145 (56.2) |
| Preventive care experience and practice environment |  | No | 113 (43.8) |
| Preventive care experience and practice environment | Teaching/lecturing experience in preventive care | Yes | 98 (38.0) |
| Preventive care experience and practice environment |  | No | 160 (62.0) |
| Preventive care experience and practice environment | Self-reported preventive care-related activity in outpatient care | Not engaged in outpatient care | 6 (2.3) |
| Preventive care experience and practice environment |  | 0–10% | 30 (11.6) |
| Preventive care experience and practice environment |  | 11–30% | 103 (39.9) |
| Preventive care experience and practice environment |  | 31–50% | 36 (14.0) |
| Preventive care experience and practice environment |  | 51–70% | 36 (14.0) |
| Preventive care experience and practice environment |  | 71–100% | 47 (18.2) |
| Preventive care experience and practice environment | Self-reported preventive care-related activity in inpatient care | Not engaged in inpatient care | 121 (46.9) |
| Preventive care experience and practice environment |  | 0–10% | 57 (22.1) |
| Preventive care experience and practice environment |  | 11–30% | 50 (19.4) |
| Preventive care experience and practice environment |  | 31–50% | 15 (5.8) |
| Preventive care experience and practice environment |  | 51–70% | 10 (3.9) |
| Preventive care experience and practice environment |  | 71–100% | 5 (1.9) |
| Preventive care experience and practice environment | Use of reminders for preventive care in regular outpatient care | Yes | 139 (53.9) |
| Preventive care experience and practice environment |  | No | 119 (46.1) |
| Preventive care resources | Preventive care resources used† | Japanese clinical guidelines | 148 (57.4) |
| Preventive care resources |  | Vaccine information website for children and adults | 112 (43.4) |
| Preventive care resources |  | United States Preventive Services Task Force recommendations | 98 (38.0) |
| Preventive care resources |  | Public institutional resources | 80 (31.0) |
| Preventive care resources |  | Kameda preventive care website | 64 (24.8) |
| Preventive care resources |  | Few or no resources used (selected option) | 37 (14.3) |
| Preventive care resources |  | Hospital manual | 34 (13.2) |
| Preventive care resources |  | Evidence-based preventive care website | 28 (10.9) |
| Preventive care resources |  | Advisory Committee on Immunisation Practices recommendations | 24 (9.3) |
| Preventive care resources |  | Other resources or free-text responses | 14 (5.4) |
| Preventive care resources | Grouped resource use | No resources only | 30 (11.6) |
| Preventive care resources |  | Any resources used | 228 (88.4) |
| Overall self-rated implementation | Overall self-rated implementation of preventive care (0–100) | Mean (standard deviation) | 48.6 (24.0); n=256 |
| Overall self-rated implementation |  | Median (interquartile range) | 50.0 (30.0–70.0); n=256 |
| Perceived need for team-based care | Team-based preventive care with other professionals | Very necessary | 106 (41.1) |
| Perceived need for team-based care |  | Necessary | 104 (40.3) |
| Perceived need for team-based care |  | Somewhat necessary | 43 (16.7) |
| Perceived need for team-based care |  | Not very necessary | 3 (1.2) |
| Perceived need for team-based care |  | Not necessary | 2 (0.8) |

*Notes:* *Percentages use N=258 as the denominator unless otherwise indicated. Specialty and resource items were multiple-response items and may sum to more than 100%. The workplace categories include two recoded free-text responses: “university branch” was grouped with university hospital, and “university basic department; regional core hospital if limited to healthcare institutions” was grouped with regional core hospital. The sex category “other, selected both, or prefer not to answer” includes respondents who selected both male and female or preferred not to answer. For grouped resource use, “no resources only” means that the respondent selected only the “few or no resources used” response option; all others were classified as “any resources used.” Self-reported preventive care-related activity does not indicate documented clinical delivery or patient receipt.*

Supplementary Table S2. Self-reported recommendation and eligibility-identification difficulty for 30 preventive services.

| **Domain** | **Preventive service** | **Valid n** | **Eligible-denominator n†** | **Mostly achieved, n/N (%)** | **Good/moderate achievement, n/N (%)** | **Unable to identify eligible patients, n/N (%)** | **No eligible patients, n/N (%)** | **Priority zone** |
| --- | --- | --- | --- | --- | --- | --- | --- | --- |
| Cancer screening | Lung cancer screening | 258 | 241 | 51/241 (21.2) | 139/241 (57.7) | 8/258 (3.1) | 17/258 (6.6) | Other |
| Cancer screening | Gastric cancer screening | 258 | 242 | 55/242 (22.7) | 163/242 (67.4) | 10/258 (3.9) | 16/258 (6.2) | Other |
| Cancer screening | Colorectal cancer screening | 258 | 244 | 70/244 (28.7) | 174/244 (71.3) | 8/258 (3.1) | 14/258 (5.4) | Other |
| Cancer screening | Breast cancer screening | 258 | 226 | 33/226 (14.6) | 96/226 (42.5) | 9/258 (3.5) | 32/258 (12.4) | Higher-priority |
| Cancer screening | Cervical cancer screening | 258 | 224 | 38/224 (17.0) | 99/224 (44.2) | 8/258 (3.1) | 34/258 (13.2) | Higher-priority |
| Vaccination | Influenza vaccination | 258 | 250 | 123/250 (49.2) | 213/250 (85.2) | 4/258 (1.6) | 8/258 (3.1) | Other |
| Vaccination | Pneumococcal vaccination | 258 | 250 | 89/250 (35.6) | 180/250 (72.0) | 5/258 (1.9) | 8/258 (3.1) | Other |
| Vaccination | COVID-19 vaccination | 258 | 250 | 88/250 (35.2) | 184/250 (73.6) | 5/258 (1.9) | 8/258 (3.1) | Other |
| Vaccination | Varicella/zoster vaccination | 258 | 246 | 64/246 (26.0) | 158/246 (64.2) | 4/258 (1.6) | 12/258 (4.7) | Other |
| Vaccination | Tetanus, diphtheria and pertussis vaccination | 258 | 217 | 40/217 (18.4) | 80/217 (36.9) | 12/258 (4.7) | 41/258 (15.9) | Higher-priority |
| Vaccination | Measles, mumps, rubella and varicella vaccination | 258 | 218 | 45/218 (20.6) | 94/218 (43.1) | 12/258 (4.7) | 40/258 (15.5) | Higher-priority |
| Vaccination | Human papillomavirus and catch-up human papillomavirus vaccination | 258 | 211 | 38/211 (18.0) | 92/211 (43.6) | 9/258 (3.5) | 47/258 (18.2) | Higher-priority |
| Vaccination | Hepatitis B vaccination | 258 | 219 | 46/219 (21.0) | 91/219 (41.6) | 17/258 (6.6) | 39/258 (15.1) | Higher-priority |
| Vaccination | Respiratory syncytial virus vaccination | 258 | 209 | 13/209 (6.2) | 25/209 (12.0) | 20/258 (7.8) | 49/258 (19.0) | Higher-priority |
| Screening | Chlamydia/gonorrhoea screening | 258 | 201 | 12/201 (6.0) | 29/201 (14.4) | 35/258 (13.6) | 57/258 (22.1) | Higher-priority |
| Screening | Hepatitis C screening | 258 | 225 | 32/225 (14.2) | 94/225 (41.8) | 27/258 (10.5) | 33/258 (12.8) | Higher-priority |
| Screening | Hypertension screening | 258 | 254 | 125/254 (49.2) | 226/254 (89.0) | 2/258 (0.8) | 4/258 (1.6) | Other |
| Screening | Dyslipidaemia screening | 258 | 255 | 121/255 (47.5) | 219/255 (85.9) | 2/258 (0.8) | 3/258 (1.2) | Other |
| Screening | Diabetes screening | 258 | 255 | 125/255 (49.0) | 218/255 (85.5) | 2/258 (0.8) | 3/258 (1.2) | Other |
| Screening | Alcohol use screening | 258 | 254 | 82/254 (32.3) | 186/254 (73.2) | 3/258 (1.2) | 4/258 (1.6) | Other |
| Screening | Abdominal aortic aneurysm screening | 258 | 238 | 18/238 (7.6) | 68/238 (28.6) | 13/258 (5.0) | 20/258 (7.8) | Higher-priority |
| Screening | Osteoporosis screening | 258 | 252 | 40/252 (15.9) | 149/252 (59.1) | 4/258 (1.6) | 6/258 (2.3) | Other |
| Screening | Anxiety screening | 258 | 250 | 28/250 (11.2) | 76/250 (30.4) | 14/258 (5.4) | 8/258 (3.1) | Higher-priority |
| Screening | Depression screening | 258 | 250 | 33/250 (13.2) | 80/250 (32.0) | 11/258 (4.3) | 8/258 (3.1) | Higher-priority |
| Screening | Domestic violence screening | 258 | 226 | 10/226 (4.4) | 25/226 (11.1) | 36/258 (14.0) | 32/258 (12.4) | Higher-priority |
| Counselling | Smoking cessation counselling | 258 | 248 | 67/248 (27.0) | 175/248 (70.6) | 3/258 (1.2) | 10/258 (3.9) | Other |
| Counselling | Obesity counselling | 258 | 253 | 61/253 (24.1) | 166/253 (65.6) | 2/258 (0.8) | 5/258 (1.9) | Other |
| Counselling | Folic acid supplementation counselling | 258 | 218 | 14/218 (6.4) | 47/218 (21.6) | 14/258 (5.4) | 40/258 (15.5) | Higher-priority |
| Counselling | Diet and exercise counselling for cardiovascular risk | 258 | 253 | 61/253 (24.1) | 170/253 (67.2) | 4/258 (1.6) | 5/258 (1.9) | Other |
| Counselling | Statin counselling for cardiometabolic risk | 258 | 252 | 71/252 (28.2) | 188/252 (74.6) | 3/258 (1.2) | 6/258 (2.3) | Other |

*Notes: †The eligible-denominator n excludes respondents who selected “no eligible patients.” “Mostly achieved” corresponds to 70–100% achieved. “Good/moderate achievement” combines 40–60% and 70–100% achieved. “Unable to identify eligible patients” and “no eligible patients” use all valid responses for each service as denominators. The priority zone was defined descriptively and exploratorily as services with unable-to-identify percentage ≥ the median across services (3.10%) and mostly-achieved percentage < the median across services (21.08%). These measures refer to physician self-reported recommendation and should not be interpreted as documented clinical delivery, patient receipt, or guideline-concordant care.*

Supplementary Table S3. Item-level percentages with 95% confidence intervals for 30 preventive services.

| **Domain** | **Preventive service** | **Mostly achieved, % (95% CI)** | **Good/moderate achievement, % (95% CI)** | **Unable to identify eligible patients, % (95% CI)** | **No eligible patients, % (95% CI)** |
| --- | --- | --- | --- | --- | --- |
| Cancer screening | Lung cancer screening | 21.2 (16.5–26.8) | 57.7 (51.4–63.7) | 3.1 (1.6–6.0) | 6.6 (4.2–10.3) |
| Cancer screening | Gastric cancer screening | 22.7 (17.9–28.4) | 67.4 (61.2–73.0) | 3.9 (2.1–7.0) | 6.2 (3.9–9.8) |
| Cancer screening | Colorectal cancer screening | 28.7 (23.4–34.7) | 71.3 (65.3–76.6) | 3.1 (1.6–6.0) | 5.4 (3.3–8.9) |
| Cancer screening | Breast cancer screening | 14.6 (10.6–19.8) | 42.5 (36.2–49.0) | 3.5 (1.8–6.5) | 12.4 (8.9–17.0) |
| Cancer screening | Cervical cancer screening | 17.0 (12.6–22.4) | 44.2 (37.8–50.7) | 3.1 (1.6–6.0) | 13.2 (9.6–17.9) |
| Vaccination | Influenza vaccination | 49.2 (43.1–55.4) | 85.2 (80.3–89.1) | 1.6 (0.6–3.9) | 3.1 (1.6–6.0) |
| Vaccination | Pneumococcal vaccination | 35.6 (29.9–41.7) | 72.0 (66.1–77.2) | 1.9 (0.8–4.5) | 3.1 (1.6–6.0) |
| Vaccination | COVID-19 vaccination | 35.2 (29.5–41.3) | 73.6 (67.8–78.7) | 1.9 (0.8–4.5) | 3.1 (1.6–6.0) |
| Vaccination | Varicella/zoster vaccination | 26.0 (20.9–31.8) | 64.2 (58.1–70.0) | 1.6 (0.6–3.9) | 4.7 (2.7–8.0) |
| Vaccination | Tetanus, diphtheria and pertussis vaccination | 18.4 (13.8–24.1) | 36.9 (30.7–43.5) | 4.7 (2.7–8.0) | 15.9 (11.9–20.8) |
| Vaccination | Measles, mumps, rubella and varicella vaccination | 20.6 (15.8–26.5) | 43.1 (36.7–49.8) | 4.7 (2.7–8.0) | 15.5 (11.6–20.4) |
| Vaccination | Human papillomavirus and catch-up human papillomavirus vaccination | 18.0 (13.4–23.8) | 43.6 (37.1–50.3) | 3.5 (1.8–6.5) | 18.2 (14.0–23.4) |
| Vaccination | Hepatitis B vaccination | 21.0 (16.1–26.9) | 41.6 (35.2–48.2) | 6.6 (4.2–10.3) | 15.1 (11.3–20.0) |
| Vaccination | Respiratory syncytial virus vaccination | 6.2 (3.7–10.3) | 12.0 (8.2–17.1) | 7.8 (5.1–11.7) | 19.0 (14.7–24.2) |
| Screening | Chlamydia/gonorrhoea screening | 6.0 (3.4–10.1) | 14.4 (10.2–20.0) | 13.6 (9.9–18.3) | 22.1 (17.5–27.5) |
| Screening | Hepatitis C screening | 14.2 (10.3–19.4) | 41.8 (35.5–48.3) | 10.5 (7.3–14.8) | 12.8 (9.3–17.4) |
| Screening | Hypertension screening | 49.2 (43.1–55.3) | 89.0 (84.5–92.3) | 0.8 (0.2–2.8) | 1.6 (0.6–3.9) |
| Screening | Dyslipidaemia screening | 47.5 (41.4–53.6) | 85.9 (81.1–89.6) | 0.8 (0.2–2.8) | 1.2 (0.4–3.4) |
| Screening | Diabetes screening | 49.0 (42.9–55.1) | 85.5 (80.6–89.3) | 0.8 (0.2–2.8) | 1.2 (0.4–3.4) |
| Screening | Alcohol use screening | 32.3 (26.8–38.3) | 73.2 (67.5–78.3) | 1.2 (0.4–3.4) | 1.6 (0.6–3.9) |
| Screening | Abdominal aortic aneurysm screening | 7.6 (4.8–11.6) | 28.6 (23.2–34.6) | 5.0 (3.0–8.4) | 7.8 (5.1–11.7) |
| Screening | Osteoporosis screening | 15.9 (11.9–20.9) | 59.1 (53.0–65.0) | 1.6 (0.6–3.9) | 2.3 (1.1–5.0) |
| Screening | Anxiety screening | 11.2 (7.9–15.7) | 30.4 (25.0–36.4) | 5.4 (3.3–8.9) | 3.1 (1.6–6.0) |
| Screening | Depression screening | 13.2 (9.6–18.0) | 32.0 (26.5–38.0) | 4.3 (2.4–7.5) | 3.1 (1.6–6.0) |
| Screening | Domestic violence screening | 4.4 (2.4–8.0) | 11.1 (7.6–15.8) | 14.0 (10.3–18.7) | 12.4 (8.9–17.0) |
| Counselling | Smoking cessation counselling | 27.0 (21.9–32.9) | 70.6 (64.6–75.9) | 1.2 (0.4–3.4) | 3.9 (2.1–7.0) |
| Counselling | Obesity counselling | 24.1 (19.3–29.7) | 65.6 (59.6–71.2) | 0.8 (0.2–2.8) | 1.9 (0.8–4.5) |
| Counselling | Folic acid supplementation counselling | 6.4 (3.9–10.5) | 21.6 (16.6–27.5) | 5.4 (3.3–8.9) | 15.5 (11.6–20.4) |
| Counselling | Diet and exercise counselling for cardiovascular risk | 24.1 (19.3–29.7) | 67.2 (61.2–72.7) | 1.6 (0.6–3.9) | 1.9 (0.8–4.5) |
| Counselling | Statin counselling for cardiometabolic risk | 28.2 (23.0–34.0) | 74.6 (68.9–79.6) | 1.2 (0.4–3.4) | 2.3 (1.1–5.0) |

*Notes: Values are percentages with 95% confidence intervals calculated using the Wilson score method. Achievement measures use the eligible denominator, excluding respondents who selected “no eligible patients.” “Unable to identify eligible patients” and “no eligible patients” use all valid responses as denominators. Confidence intervals are descriptive and were not used for formal pairwise comparisons between services.*

Supplementary Table S4. Quadrant classification for the two-axis priority map.

| **Quadrant** | **No. of services** | **Preventive services** |
| --- | --- | --- |
| Higher difficulty / lower achievement (eligibility-identification priority zone) | 14 | Breast cancer screening; Cervical cancer screening; Tetanus, diphtheria and pertussis vaccination; Measles, mumps, rubella and varicella vaccination; Human papillomavirus and catch-up human papillomavirus vaccination; Hepatitis B vaccination; Respiratory syncytial virus vaccination; Chlamydia/gonorrhoea screening; Hepatitis C screening; Abdominal aortic aneurysm screening; Anxiety screening; Depression screening; Domestic violence screening; Folic acid supplementation counselling |
| Higher difficulty / higher achievement | 3 | Lung cancer screening; Gastric cancer screening; Colorectal cancer screening |
| Lower difficulty / lower achievement | 1 | Osteoporosis screening |
| Lower difficulty / higher achievement | 12 | Influenza vaccination; Pneumococcal vaccination; COVID-19 vaccination; Varicella/zoster vaccination; Hypertension screening; Dyslipidaemia screening; Diabetes screening; Alcohol use screening; Smoking cessation counselling; Obesity counselling; Diet and exercise counselling for cardiovascular risk; Statin counselling for cardiometabolic risk |

*Notes: Higher difficulty was defined as unable-to-identify percentage ≥ 3.10%. Higher achievement was defined as mostly-achieved percentage ≥ 21.08%. The higher-difficulty/lower-achievement quadrant corresponds to the descriptive eligibility-identification priority zone. Quadrants are based on medians across services and should not be interpreted as definitive rankings of clinical importance.*

Supplementary Table S5. Sensitivity analyses for alternative priority-zone definitions.

| **Scenario** | **Definition** | **Difficulty threshold (%)** | **Mostly-achieved threshold (%)** | **No. of services classified** | **Services classified** |
| --- | --- | --- | --- | --- | --- |
| Primary median-based definition | Unable-to-identify percentage ≥ median across all 30 services; mostly-achieved percentage < median across all 30 services | 3.10 | 21.08 | 14 | Breast cancer screening; Cervical cancer screening; Tetanus, diphtheria and pertussis vaccination; Measles, mumps, rubella and varicella vaccination; Human papillomavirus and catch-up human papillomavirus vaccination; Hepatitis B vaccination; Respiratory syncytial virus vaccination; Chlamydia/gonorrhoea screening; Hepatitis C screening; Abdominal aortic aneurysm screening; Anxiety screening; Depression screening; Domestic violence screening; Folic acid supplementation counselling |
| Fixed cut-offs A | Unable-to-identify percentage ≥3%; mostly-achieved percentage <20% | 3.00 | 20.00 | 12 | Breast cancer screening; Cervical cancer screening; Tetanus, diphtheria and pertussis vaccination; Human papillomavirus and catch-up human papillomavirus vaccination; Respiratory syncytial virus vaccination; Chlamydia/gonorrhoea screening; Hepatitis C screening; Abdominal aortic aneurysm screening; Anxiety screening; Depression screening; Domestic violence screening; Folic acid supplementation counselling |
| Fixed cut-offs B | Unable-to-identify percentage ≥5%; mostly-achieved percentage <20% | 5.00 | 20.00 | 7 | Respiratory syncytial virus vaccination; Chlamydia/gonorrhoea screening; Hepatitis C screening; Abdominal aortic aneurysm screening; Anxiety screening; Domestic violence screening; Folic acid supplementation counselling |
| Rank-based upper-tertile difficulty and lower-tertile achievement | Services in the highest 10 for difficulty identifying eligible patients and the lowest 10 for mostly achieved recommendation | 4.65 | 15.87 | 7 | Respiratory syncytial virus vaccination; Chlamydia/gonorrhoea screening; Hepatitis C screening; Abdominal aortic aneurysm screening; Anxiety screening; Domestic violence screening; Folic acid supplementation counselling |
| Median difficulty and lower-quartile achievement | Unable-to-identify percentage ≥ median; mostly-achieved percentage < lower quartile | 3.10 | 14.32 | 8 | Respiratory syncytial virus vaccination; Chlamydia/gonorrhoea screening; Hepatitis C screening; Abdominal aortic aneurysm screening; Anxiety screening; Depression screening; Domestic violence screening; Folic acid supplementation counselling |
| Thresholds recalculated  after excluding selected sex- or age-specific services | Medians recalculated after excluding breast, cervical, human papillomavirus, abdominal aortic aneurysm and folic acid services; classification limited to remaining services | 1.94 | 24.11 | 11 | Lung cancer screening; Gastric cancer screening; Tetanus, diphtheria and pertussis vaccination; Measles, mumps, rubella and varicella vaccination; Hepatitis B vaccination; Respiratory syncytial virus vaccination; Chlamydia/gonorrhoea screening; Hepatitis C screening; Anxiety screening; Depression screening; Domestic violence screening |
| Strict fixed cut-offs | Unable-to-identify percentage ≥5%; mostly-achieved percentage <15% | 5.00 | 15.00 | 7 | Respiratory syncytial virus vaccination; Chlamydia/gonorrhoea screening; Hepatitis C screening; Abdominal aortic aneurysm screening; Anxiety screening; Domestic violence screening; Folic acid supplementation counselling |

*Notes: These classifications are descriptive and exploratory. Fixed cut-offs, rank-based thresholds, and recalculated medians after excluding selected sex- or age-specific services were used to examine whether the priority-zone classification changed under alternative definitions. The sensitivity analysis excluding selected sex- or age-specific services recalculated medians after excluding breast cancer screening, cervical cancer screening, human papillomavirus and catch-up human papillomavirus vaccination, abdominal aortic aneurysm screening, and folic acid supplementation counselling; classification was then limited to the remaining services. These analyses should not be interpreted as validating a definitive threshold for priority.*

Supplementary Table S6. Exploratory descriptive comparisons of respondent-level implementation scores.

| **Characteristic** | **Category** | **n** | **Composite implementation score, mean (SD), %** | **Overall self-rated preventive care score, mean (SD)** |
| --- | --- | --- | --- | --- |
| Structured training in preventive care | Yes | 145 | 61.6 (22.8) | 56.6 (20.8) [n=143] |
| Structured training in preventive care | No | 113 | 46.7 (26.1) | 38.5 (23.9) |
| Teaching/lecturing experience in preventive care | Yes | 98 | 62.0 (24.0) | 57.9 (22.5) [n=96] |
| Teaching/lecturing experience in preventive care | No | 160 | 50.8 (25.3) | 43.0 (23.1) |
| Use of reminders for preventive care in regular outpatient care | Yes | 139 | 61.9 (24.0) | 56.8 (22.5) |
| Use of reminders for preventive care in regular outpatient care | No | 119 | 47.1 (24.6) | 38.8 (21.9) [n=117] |
| Resource use | No resources only | 30 | 40.2 (28.2) | 35.1 (22.5) [n=29] |
| Resource use | Any resources used | 228 | 57.0 (24.4) | 50.3 (23.6) [n=227] |
| Years in practice | <5 years | 19 | 41.7 (24.8) | 32.9 (18.2) |
| Years in practice | 5 to <10 years | 21 | 53.4 (23.3) | 47.4 (20.2) |
| Years in practice | 10 to <20 years | 77 | 53.4 (24.6) | 50.0 (21.9) |
| Years in practice | 20 to <30 years | 80 | 60.5 (23.9) | 54.5 (22.8) |
| Years in practice | ≥30 years | 61 | 54.7 (27.8) | 44.2 (28.0) [n=59] |
| Primary workplace | University hospital | 42 | 53.1 (27.1) | 41.3 (22.2) |
| Primary workplace | Regional core hospital | 34 | 52.4 (24.7) | 46.5 (23.8) |
| Primary workplace | Small- to medium-sized hospital | 69 | 49.0 (24.6) | 45.4 (24.3) |
| Primary workplace | Clinic | 108 | 60.1 (24.5) | 53.3 (23.6) [n=106] |
| Primary workplace | Other | 5 | 64.2 (29.9) | 68.0 (17.9) |
| Age group | ≤34 years | 33 | 45.6 (24.4) | 38.5 (20.7) |
| Age group | 35–44 years | 60 | 56.3 (22.0) | 51.8 (20.3) |
| Age group | 45–54 years | 82 | 57.5 (25.6) | 51.7 (23.6) |
| Age group | 55–64 years | 61 | 57.8 (26.3) | 49.8 (27.1) [n=59] |
| Age group | ≥65 years | 22 | 49.1 (29.5) | 40.5 (26.1) |

*Notes: Comparisons are descriptive and exploratory and should not be interpreted causally. The composite implementation score was calculated for each respondent as the percentage of preventive services rated “moderately achieved” or “mostly achieved,” excluding services for which the respondent reported “no eligible patients” and retaining “unable to identify eligible patients” as not achieved. Non-numeric responses were excluded from summaries of the overall self-rated preventive care score. No adjustment for multiple comparisons was applied because these analyses were exploratory.*

Supplementary Table S7. Exploratory multivariable analysis of the respondent-level composite implementation score.

| **Variable and comparison** | **Coefficient, percentage points** | **95% confidence interval** | **P value** |
| --- | --- | --- | --- |
| Structured training in preventive care: Yes vs no | 10.0 | 3.2 to 16.7 | 0.004 |
| Teaching/lecturing experience: Yes vs no | 3.3 | -3.0 to 9.6 | 0.302 |
| Reminder use: Yes vs no | 9.2 | 1.8 to 16.7 | 0.016 |
| Any preventive care resource used vs no resources only | 9.1 | -2.9 to 21.1 | 0.137 |
| Age group: 35–44 years vs ≤34 years | 5.8 | -4.8 to 16.4 | 0.282 |
| Age group: 45–54 years vs ≤34 years | 8.7 | -2.3 to 19.6 | 0.121 |
| Age group: 55–64 years vs ≤34 years | 12.7 | 0.7 to 24.7 | 0.038 |
| Age group: ≥65 years vs ≤34 years | 7.7 | -8.1 to 23.5 | 0.339 |
| Sex: female vs male | 2.0 | -4.9 to 8.9 | 0.577 |
| Sex: other, selected both, or prefer not to answer vs male | 5.8 | -27.6 to 39.2 | 0.733 |
| Primary workplace: university hospital vs clinic | -0.7 | -10.5 to 9.1 | 0.889 |
| Primary workplace: regional core hospital vs clinic | -8.1 | -18.3 to 2.0 | 0.115 |
| Primary workplace: small- to medium-sized hospital vs clinic | -8.3 | -15.4 to -1.1 | 0.024 |
| Primary workplace: other vs clinic | -5.6 | -38.3 to 27.2 | 0.739 |

*Notes: The outcome was the composite implementation score, scaled from 0 to 100 percentage points. Coefficients represent adjusted percentage-point differences. The model included all variables shown in the table. Confidence intervals and P values use HC3 heteroscedasticity-robust standard errors. N=258; R^2^=0.194; adjusted R^2^=0.147. Results are exploratory and should not be interpreted causally.*

Supplementary Table S8. Themes from free-text comments with anonymised illustrative quotations.

| **Theme** | **No. of comments coded†** | **Summary** | **Illustrative quotation translated from Japanese** |
| --- | --- | --- | --- |
| Financing, reimbursement and incentives | 24 | Respondents described insurance coverage, reimbursement, out-of-pocket costs and incentives as implementation conditions. | “If preventive care is to be emphasised, there should be mechanisms such as insurance coverage or tax deductions for preventive care costs.” |
| Team-based care and interprofessional roles | 16 | Comments emphasised roles for nurses, pharmacists, dietitians, physical therapists and other professionals rather than physician-only delivery. | “It is difficult for physicians alone; preventive care needs to be approached from multiple angles by a team.” |
| Education, training, guidelines and resources | 20 | Respondents asked for practical guidance, educational opportunities, teaching materials and evidence summaries suited to Japanese practice. | “In general hospitals there are few opportunities to learn preventive care systematically; accessible information and educational materials are needed.” |
| Patient and public awareness, outreach and misinformation | 19 | Comments highlighted limited patient awareness, vaccine hesitancy, misinformation and the need for public education. | “In an era of misinformation on social media, accurate information from healthcare professionals and public outreach are important.” |
| Systems, reminders and workflow integration | 15 | Respondents described needs for checklists, electronic records, prompts, registries, recall systems and links between clinical care and health checks. | “A system that immediately shows whether preventive care has been addressed for each patient would be helpful.” |
| Scope, definitions, ethics and overmedicalisation | 8 | Some comments questioned the scope, definition and limits of preventive care, including potential overuse and withdrawal criteria. | “It would be helpful to have criteria for when to stop screening, such as up to what age cancer screening should be continued.” |
| Time, feasibility and workload | 14 | Comments described the time and effort required to explain prevention and integrate it into busy consultations. | “Preventive care is important, but there are time constraints in outpatient practice.” |
| Policy, administrative coordination and primary care role | 20 | Respondents noted responsibilities of primary care, local government, medical associations and health-system policy. | “It would be ideal if primary care physicians could provide vaccinations, screening and other preventive care with public support.” |

*Notes: Free-text comments were available from 65 respondents. Three comments indicating no additional comment were excluded, leaving 62 substantive comments. Free-text comments were coded thematically by multiple authors and refined through discussion among the author group. Comments could be assigned to multiple themes, so counts may sum to more than the number of substantive comments. Quotations are translated from Japanese and lightly edited for clarity while preserving meaning.*
